# Rhythmic complexity and predictive coding: a novel approach to modeling rhythm and meter perception in music

**DOI:** 10.3389/fpsyg.2014.01111

**Published:** 2014-10-01

**Authors:** Peter Vuust, Maria A. G. Witek

**Affiliations:** ^1^Center of Functionally Integrative Neuroscience, Aarhus University HospitalAarhus, Denmark; ^2^Royal Academy of MusicAarhus/Aalborg, Denmark

**Keywords:** rhythm, meter, rhythmic complexity, predictive coding, pleasure

## Abstract

Musical rhythm, consisting of apparently abstract intervals of accented temporal events, has a remarkable capacity to move our minds and bodies. How does the cognitive system enable our experiences of rhythmically complex music? In this paper, we describe some common forms of rhythmic complexity in music and propose the theory of predictive coding (PC) as a framework for understanding how rhythm and rhythmic complexity are processed in the brain. We also consider why we feel so compelled by rhythmic tension in music. First, we consider theories of rhythm and meter perception, which provide hierarchical and computational approaches to modeling. Second, we present the theory of PC, which posits a hierarchical organization of brain responses reflecting fundamental, survival-related mechanisms associated with predicting future events. According to this theory, perception and learning is manifested through the brain’s Bayesian minimization of the error between the input to the brain and the brain’s prior expectations. Third, we develop a PC model of musical rhythm, in which rhythm perception is conceptualized as an interaction between what is heard (“rhythm”) and the brain’s anticipatory structuring of music (“meter”). Finally, we review empirical studies of the neural and behavioral effects of syncopation, polyrhythm and groove, and propose how these studies can be seen as special cases of the PC theory. We argue that musical rhythm exploits the brain’s general principles of prediction and propose that pleasure and desire for sensorimotor synchronization from musical rhythm may be a result of such mechanisms.

## INTRODUCTION

Music can move us, both emotionally and corporeally. It can send shivers down our spines and make us tap our feet in time with the beat. How does the brain facilitate the rich and complex experiences we have of rhythm in music? Here, we propose the theory of predictive coding (PC) as a framework for understanding the ways in which complex rhythms are processed in the brain and discuss why we derive pleasure from rhythm in music. First, we point to the theories of rhythm and meter which allow for hierarchical and computational modeling. Second, we present the theory of PC, which posits a hierarchical organization of neural functioning, reflecting fundamental mechanisms associated with predicting future events. The theory puts forward that perception and learning occurs in a recursive Bayesian process by which the brain tries to minimize the error between the input and the brain’s expectation. Third, we view rhythm perception in light of this theory as an interaction between what is heard (“rhythm”) and the brain’s anticipatory model (“meter”). We describe the experience of rhythm in music as depending on the degree of tension or discrepancy between rhythm and meter. Finally, we review some empirical studies of different forms of tension between rhythm and meter – syncopation, polyrhythm and groove – and propose that these can be seen as special cases of PC. Our examples illustrate a number of fundamental principles of its mechanisms; the effects of prior experience, model comparison, and the relationship between prediction error and affective and embodied responses.

## HIERARCHICAL MODELS OF RHYTHM AND METER

Theories of rhythmic perception often contrast rhythm with meter. Broadly, *rhythm* is a pattern of discrete durations and is largely thought to depend on the underlying perceptual mechanisms of grouping (Fraisse, 1963, 1982, 1984; Clarke, 1999). *Meter*, again broadly, is the temporal framework according to which rhythm is perceived. More specifically, as defined by London (2012, p. 4): “meter involves our initial perception as well as subsequent anticipation of a series of beats that we abstract from the rhythmic surface of the music as it unfolds in time.” At the most basic level, the perception of meter involves a sense of pulse, i.e., a pattern of beats at isochronously spaced intervals (Honing, 2012, 2013). When such beats are hierarchically differentiated into strong and weak accents, it is thought that we perceive meter (Lerdahl and Jackendoff, 1983; London, 2012). Because of its hierarchical nature, meter allows for rhythmic expectations in music (Large and Kolen, 1994; Jones, 2009; Ladinig et al., 2009; Rohrmeier and Koelsch, 2012). In other words, meter provides the listener with an expectancy structure underlying the perception of music according to which each musical time-point encompasses a conjoint perception of time and salience.

However, there are instances in which this sharp distinction between rhythm (perceived) and meter (induced) becomes blurred. Teki et al. (2011a,b) distinguish between duration-based and beat-based timing associated with the coordination and temporal patterning of body-movements (see also McAuley and Jones, 2003; Grahn and McAuley, 2009). In the former, time is organized sequentially and relies on absolute intervallic relationships between discrete events. In the latter, time intervals are organized relative to overall temporal regularity. In other words, beat-based rhythms subserve and enable hierarchical meter. In such cases, the rhythm is perceived as reflecting its underlying metric organization. This is the most common form of timing perceived in music. As we shall see, the theory of PC offers a way of understanding what goes on in our brains when the beats do not seem to uniformly correspond to one single regularity framework.

**FIGURE 1:**
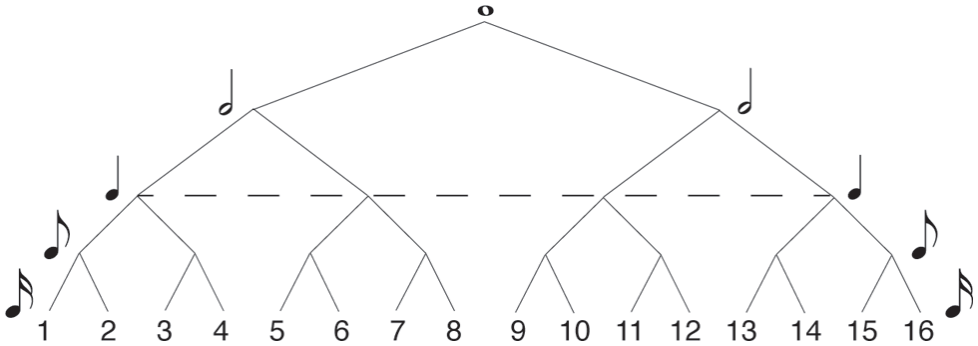
**Hierarchical model of 4/4 meter.** Each metric level (or value) is recursively subdivided into equally spaced parts (or values) at the level below, determining the metric salience of positions within the metric framework. The higher the level in the hierarchy, the more salient the position in the meter. Numbers designate serial positions within the meter, at 16th note resolution. The dashed line specifies the level of the tactus.

In formal music-theory terms, meter is often specified in the time signature traditionally given at the beginning of a musical score. Some common time signatures in Western tonal and metric music are 4/4, 2/4, and 3/4. In these time signatures, the first digit indicates the number of pulses in the bar, and the second indicates their durational value. Hierarchical meters are organized by the recursive subdivision of each metric level, both above and below the main pulse (or tactus). **Figure 1** shows how metric levels and their corresponding note durations are organized hierarchically in a 4/4 bar. In 4/4, the metric hierarchy is duple. Each level – from the whole-note level to the level of 16th notes ^1^ – is recursively subdivided into two equal parts. The ways of subdividing each metrical level vary in other time signatures, such as compound meters like 6/8 in which the duple tactus is divided into three at the eighth note level, or more complex meters like 5/4 in which the tactus is quintuple, but other subdivisions are duple. However, the time signature of a given piece can often be notated in more than one way, and the subjective experience of its meter may be at odds with its formal time signature. There is, more generally, greater disagreement about the perceptual definition of meter, compared to formal metric categories. While most agree on the particular salience of the tactus (Parncutt, 1994; Agawu, 2003; Jones, 2009; Honing, 2012), the extent of hierarchical differentiation of pulse sequences beyond the tactus (i.e., at higher or lower levels) is still unknown (Ladinig et al., 2009; Witek et al., in press). Lerdahl and Jackendoff (1983) have proposed a highly hierarchical theory of meter, in which rhythm perception is thought to be underpinned by a metric framework organized in a tree-like structure (similar to that of **Figure 1**). This hierarchical structure is derived from the representation of the musical input which interacts with a small number of top-down cognitive rules. Similar tree-like organizations of meter feature in Longuet-Higgins and Lee’s (1984) computational model of rhythmic syncopation. Here, each metric level is associated with a metric weight – the higher the level, the more salient its metric values. Although Palmer and Krumhansl (1990) found such highly hierarchical structures reflected in the rhythmic perception of musicians, more recent studies have found it difficult to empirically demonstrate that listeners’ (both musicians and non-musicians) metric hierarchies extend beyond the salience of the downbeat (Ladinig et al., 2009; Song et al., 2013; Witek et al., in press).

In another influential model of meter, dynamic attending theory (DAT), different metric levels are also thought to vary in salience in relation to each other, but such hierarchical relationships are seen as much more dynamic, adaptive and flexible (Large and Kolen, 1994; Large and Jones, 1999; Barnes and Jones, 2000; Jones, 2004, 2009). Originally proposed as a model for temporal expectations more generally (Large and Jones, 1999; Barnes and Jones, 2000; Jones, 2004, 2009), DAT has since been specifically applied to music (Clayton et al., 2004; Molnar-Szakacz and Overy, 2006; Phillips-Silver et al., 2010; London, 2012; Trost and Vuilleumier, 2013). DAT posits that metric frameworks are perceived in rhythm by way of entrainment. The listener’s attention is captured and driven by the periodicities (or oscillations) in the rhythmic pattern, and the experience of metric accents corresponds to the relative strength of attention directed toward each rhythmic event, distributed hierarchically and isochronously across a rhythmic measure. In this way, meter emerges as a consequence of the reciprocal relationship between external periodicities and internal attending processes. Although bottom-up and top-down processes are acknowledged in both theories (albeit not explicitly in Lerdahl and Jackendoff, 1983), Lerdahl and Jackendoff (1983) focus on final-state representations of meter, while DAT (Large and Kolen, 1994; Large and Jones, 1999; Barnes and Jones, 2000; Jones, 2004, 2009) treats bottom-up and top-down processing simultaneously and is more concerned with the dynamic process underlying meter perception. As will soon become clear, such equal emphasis on bottom-up and top-down is one aspect that DAT shares with PC.

It is becoming increasingly common to model metrical perception using wholly computational models (e.g., Desain and Honing, 1999; Temperley and Sleator, 1999; Dixon, 2001; Margulis and Beatty, 2008; Volk, 2008). Temperley’s (2004, 2007, 2009, 2010) influential model of rhythm and meter uses “Bayes’ rule,” a computational theorem that allows the calculation of probabilities of certain observations based on prior statistical information. Through a generative process similar to that proposed by Lerdahl and Jackendoff (1983), Temperley proposes that meter is inferred from the probabilities of different patterns of regularity generated by a given rhythmic input. In one study (Temperley, 2010), he tested the performance of six probabilistic models of meter, calculated using Bayes’ rule of probability, on two corpuses of music; the Essen Folk Song Collection (Schaffrath, 1995) and a collection of string quartets by Hayden and Mozart. The Bayesian model allowed Temperley to draw conclusions about how well a sample of data (e.g., a rhythmic pattern) fits with other samples of the same type of data more generally (a model of rhythm or meter). As will become clear below, such Bayesian approaches can also be seen as the basis of perceptual processing more generally, from the level of individual neurons, to subjective affective experience.

## PREDICTIVE CODING

The idea that perception can be modeled as a Bayesian process is the point of departure for a relatively novel way of understanding fundamental brain function. The theory of PC tries to explain how areas in the brain share and integrate information. It was first proposed by Friston (2002, 2005), but preceded by several similar theories about fundamental brain processing centered on prediction (Mumford, 1992, 1994; Rao and Ballard, 1999). Via Bayesian inference, the brain predicts the causes and sources of its internal states from the actual sensory input as compared with previous “knowledge,” accumulated through experience (Friston, 2005). In this way, the brain is a “hypothesis-tester” and its goal is to “explain away” prediction error by adapting its *a priori* predictions. Mathematically speaking, it uses Bayes’ rule recursively (i.e., from level to level in the nested neural networks) to infer the probability of its hypothesis, given the equation p(a|b) = p(b|a)^∗^p(a)/p(b), where b is the input and a is the hypothesis (see Temperley, 2007 for a very accessible and music-oriented explanation of Bayes’ theorem). Note that Bayesian inference is assumed to take place at every level of brain processing so that higher levels of processing provide priors for lower levels, thus creating nested and hierarchical links across the entire brain. The PC theory assumes a multi-level cascade of processing at different time-scales, in which each level attempts to predict the activity at the level below it via backward connections. The higher-level predictions act as priors for the lower-level processing (so-called “empirical Bayes,” Robbins, 1956). These priors are influenced by previous experience and culture (Roepstorff et al., 2010), often termed hyper-priors (Friston, 2008). However, it is not only experiences from the lifetime scale that affect the process; more short-term priors also influence predictions that are made on a moment-to-moment basis. For example, while the experience of a metrically complex rhythmic pattern will depend on whether one has been exposed to such rhythms in playing (Vuust et al., 2012b) or listening (Kalender et al., 2013), the perception of it will also depend on how frequently this pattern is featured within the current musical context (Huron, 2006).

The relationship between bottom-up (input) and top-down (prediction) processes is entirely mutually dependent, and the comparison between them is essential to the system, since a variety of environmental causes can theoretically result in similar sensory input (e.g., a cat vs. an image of a cat). The top-down models provide the brain with context-sensitive ways of selecting the correct interpretation of the incoming information. The predictive models continuously predict the causal relationship between sensory input and environmental events. In changing environments, the models are gradually updated (as a result of the bottom-up prediction error) to maximize the correspondence between the sensory input and the predictions, and minimize prediction error. In this way, the causes of sensations are not solely backtracked from the sensory input, but also inferred and anticipated based on contextual cues and previous sensations. Thus, perception is a process that is mutually manifested between the perceiver and the environment, reflecting the bottom-up/top-down reciprocity that is also central to DAT, as mentioned above.

According to PC, the process of comparing input to predictions occurs hierarchically at every level of processing, from the interaction between individual neurons, to communication between large populations of neurons (i.e., brain areas or networks). Furthermore, there are both forward and backward projections between the different layers in the system (Rao and Ballard, 1999). Using a simplified physiological model of PC we can assume that mainly superficial layers in the cortex, rich in pyramidal cells, are responsible for forwarding prediction error upward in the system (driving), whereas mainly modulatory feedback connections from deeper layers provide predictions from higher cortical areas to suppress prediction errors at the lower levels (Bastos et al., 2012). In this way, specific neuronal populations are associated with specific computational roles, disclosing the correspondence between the microcircuitry of the cortical column and the connectivity implied by PC. Hence, at any given level, the input is compared with the prediction from the level above (backward projection). If there is any discrepancy between the two, the difference, i.e., the *prediction error*, is fed forward to the next level (forward projections). At the original level, predictions are changed to comply with the input. Depending on the degree of violation, the brain does this by either updating the model, or changing the way it samples information from the environment. This dynamic and continuous updating of models and sampling methods is the basis for the system’s adaptive learning and plasticity (Friston, 2003). When predictions change, the connectivity between the neurons is believed to change accordingly. According to PC, the brain’s task is to minimize prediction error and its ultimate goal is to attain a fully predicted representation of the world. This results in a system which is highly efficient, since only the prediction error and no redundant (predicted) information needs to be processed. This is a key component of PC that sets it apart from previous theories of prediction and Bayesian inference. The only information that needs to be communicated “upward” is the prediction error, making it a kind of proxy (Feldman and Friston, 2010) for sensory information itself.

Predictive coding is notoriously difficult to prove by imaging or recording in the human brain due to the spatial and temporal limitations of the available methods, such as functional magnetic resonance imaging (fMRI), positron emission tomography (PET), electroencephalography (EEG) and magnetoencephalography (MEG). Thus, it remains a theory whose empirical validation is yet to be completed. Nonetheless, PC is supported by recent developments in our understanding of brain physiology (see Bastos et al., 2012 for a summary), and this physiological implementation of PC conforms to what we know about error processing in the brain. One particularly well-understood neural marker for error processing (or change detection), which has been frequently employed in auditory and music experiments, is the mismatch negativity (MMN) as recorded with EEG/MEG. The neuronal origins of MMN share physiological features with the error-units suggested by PC, originating in dense superficial layers of the auditory cortices.

Furthermore, there are recent behavioral studies indicating that humans act as rational Bayesian estimators, in perception and action, across different domains (Körding et al., 2007; Yu, 2007; Berniker and Körding, 2008). Recent research into rhythmic tapping is closely related to such studies: Konvalinka et al. (2010) showed that when two participants tap together (instructed to keep the tempo and synchronize with each other), they adapt to each other at a tap-by-tap basis, meaning that each tapper speeds up when the other has been faster on the last tap, and slows down if the other has been slower. In other words, interactive tappers seem to be trying to minimize prediction error at a microtemporal level (although the authors do not strictly use PC in interpreting their results). More recently, Elliott et al. (2014) provided evidence that, compared to alternative models, Bayesian modeling could better account for the behavior of participants instructed to “tap in time” with two irregular metronomes separated by a lag, suggesting that humans exploit Bayesian inference to control movement timing in situations where the underlying beat structure of auditory signals needs to be resolved (i.e., beat-based timing). Specifically, compared with models based exclusively on separation or integration of cues, the Bayesian inference model better predicted participants’ behavior and motor timing errors, since it infers the choice of separation vs. integration based on the likelihood of the onsets of the competing cues and the prior expectation of the beats’ occurrence. Cicchini et al. (2012) found that the accuracy with which percussionists were able to reproduce isolated timing intervals (duration-based timing) was more successfully predicted using a Bayesian model whose prior was estimated from statistical information about mean and standard deviation of interval distribution, compared with a model which ignored such priors. It should be noted, however, that if the system from the outside looks as if it applies Bayesian inference, it does not necessarily mean that its intrinsic mechanisms are guided by Bayesian computational principles (Maloney and Mamassian, 2009). Furthermore, even if the architecture of the brain is governed by Bayes’ rule, it does not mean that all behavior and conscious experience should reflect it (Clark, 2013). Human rhythmic behavior and sensorimotor synchronization, both in musical (e.g., Large, 2000; Repp, 2005; Keller, 2008; Repp and Keller, 2008; Schogler et al., 2008; Pecenka and Keller, 2011; Demos et al., 2012) and non-musical domains (Lee, 1998; Large and Jones, 1999; Mayville et al., 2001; Schmidt et al., 2011), have been theorized in a number of ways. As mentioned, DAT has proved a particularly useful framework for understanding dynamic temporal and motor processes. We are not claiming that this and other theories are wrong, but rather that PC provides a broader framework according to which they can be understood. The findings of DAT and other compatible research build a strong case for PC, and as we shall see below, several examples of perception of rhythmic complexity in music seem to support it as well.

Predictive coding has received wide recognition in the cognitive sciences and remains a frequently discussed topic (Rao and Ballard, 1999; Friston, 2010; Brown et al., 2011; Clark, 2013). Recently, cognitive philosopher Clark (2013) proposed that the theory could provide the much sought after “grand unifying theory” of cognition. Advocating embodied approaches to cognition (Clark and Chalmers, 1998; Clark, 2008), PC appeals to Clark particularly due to the close relationship it posits between action and perception (Friston et al., 2010). By emphasizing what he calls “action-oriented predictive processing,” he asserts that action follows the same computational strategies as perception, namely Bayesian inference. The only difference is that in motor systems, the perceiver’s own movements and active engagement with the environment constitute the prediction error minimization (Friston, 2003). Ultimately, action-oriented predictive processing is a way to mold the world and actively elicit, via body-movement, the brain’s sensory input. Thus, action and perception work together in a loop to selectively sample and actively sculpt the environment, a principle that has important commonalities with theories of situated and embodied cognition (Verschure et al., 2003; Leman, 2007; Clark, 2008). Furthermore, Clark (2013) suggests that such a principle easily allows for extensions into theories of social action and cultural environments. He also notes how interpersonal music-making can be seen as a form of multi-agent cooperation to collectively shape sensory input through sensorimotor synchronization (Molnar-Szakacz and Overy, 2006; Repp and Keller, 2008; Overy and Molnar-Szakacs, 2009; Phillips-Silver et al., 2010). But to what extent can PC help us understand rhythm and meter perception at a more basic level? Can the way we perceive and produce complex rhythm in music be seen as a Bayesian process? And to what extent can our affective responses to rhythm in music be seen as a result of predictive mechanisms? In the following discussion we will use special cases of rhythmic complexity in music to demonstrate how the relationship between rhythm and meter, one of the most fundamental premises for music perception, is an expression of input vs. model, bottom-up vs. top-down, action-perception loops, and Bayesian PC.

## PREDICTIVE CODING IN MUSICAL RHYTHM

The principles of PC align closely with the statistical learning account of melodic perception proposed by Pearce and Wiggins (2006) and Pearce et al. (2010). Their notion that initial neuronal error messages are followed by synchronized activity in various brain areas in response to low-probability sequences corresponds to the local prediction error at a low hierarchical level posited in PC. The ensuing neural synchronization across various brain areas is analogous with the integration of new information into the models at higher hierarchical layers. Recently, Vuust and Frith (2008), Vuust et al. (2009), and Gebauer et al. (2012) have suggested that PC can provide a useful framework for understanding music perception in general, and rhythm perception in particular.

**FIGURE 2:**
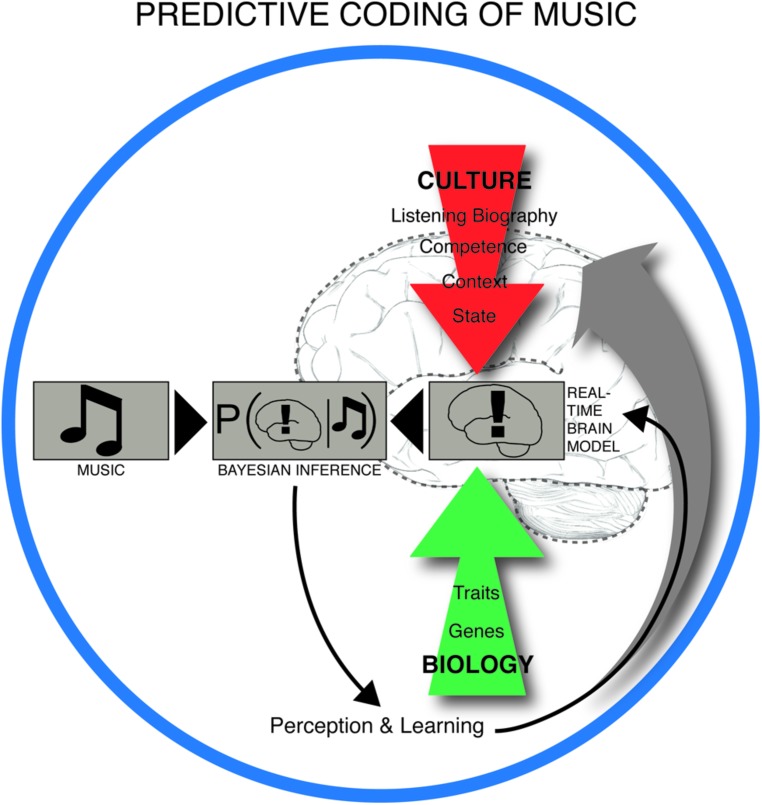
**Predictive coding of music.** The experience and learning of music takes place in a dynamic interplay between anticipatory structures in music, such as the build-up and relief of tension in rhythm, melody, harmony, form and other intra-musical features on one side, and the predictive brain on the other. The real time brain model is dependent on cultural background, personal listening history, musical competence, context (e.g., social environment), brain state (including attentional state and mood), and innate biological factors. The brain is constantly trying to minimize the discrepancy between its interpretation model and the musical input by iteratively updating the real time brain model (or prior) by weighting this model with the likelihood (musical input) through Bayes’ theorem. This leads to a constantly changing musical experience and long-term learning.

Central to this claim is that meter constitutes a key predictive model for the musical brain, shaped by statistical learning, and repeatedly challenged by the sensory input from rhythmic patterns in the actual music. Perception of rhythm is thus heavily dependent on the metrical prior. Brochard et al. (2003) have demonstrated the automaticity of this process in a remarkably simple experiment. They showed that listening to an undifferentiated metronome pattern caused the brain to register some beats as automatically more salient than others. Specifically, it arranged them into an alternating strong-weak pattern, i.e., according to duple meter. In PC terms, the brain interpreted the input – in this case the metronomic beats – based on its own predictions. Duple meters are statistically the most common in Western metric music (Temperley, 2010) and are embodied in human locomotion (Sadeghi et al., 2000). Thus the brain maximizes successful prediction by expecting rhythms to be duple (as opposed to, e.g., triple or compound). These predictive brain mechanisms are dependent on long-term learning, familiarity with a particular piece of music, deliberate listening strategies and short-term memory during listening (Altenmuller, 2001). In this way, neural structures underlying musical expectation are influenced by culture, personal listening history, musical training, mood, listening situation, and biology (**Figure 2**).

The proposed hierarchical processing in PC makes the theory particularly illustrative of the mechanisms behind meter perception in music. Although the extent of the hierarchical differentiation between pulse levels in meter is debated (Witek et al., in press), one cannot define meter without acknowledging at least *some* degree of hierarchy (e.g., between the whole-note level and the subsequent levels, as evidenced by the increased metric salience of the downbeat, Ladinig et al., 2009; Song et al., 2013; Witek et al., in press). For meter perception, PC can explain how lower levels, e.g., events at the eighth-note level, provide metric information about the whole-note level and the salience of the downbeat (feed forward). At the same time, the whole-note level, as marked by the most salient beat, the downbeat, provides a metric framework according to which the eighth-notes at the lower level are heard (feed back). This PC way of understanding metric hierarchies emphasizes the mutual relationship between bottom-up and top-down processes.

The influence of top-down processes has been demonstrated in neuroimaging studies of rhythm and beat perception. During passive listening to rhythms (i.e., with no direct priming for body-movement), Chen et al. (2008a) found activations of cortical secondary motor areas, such as the supplementary motor area and premotor area, indicating inherent coupling in the brain between action and perception. Grahn and Rowe (2009) showed that connections between such secondary motor areas and the auditory cortex were more strongly coupled during duration-beat (rhythms whose underlying beat was induced through varying rhythmic interval) than during volume-beat (rhythms whose underlying beat was induced through alternating dynamics). This suggests that secondary motor areas increase their feedback to primary sensory areas during meter perception. Similar findings were reported by Bengtsson et al. (2009), who observed parametric modulation of activity in cortical motor areas as a function of stimulus predictability (isochronous, metric or non-metric), suggesting that these areas are involved in prediction. In accordance with previous research (Raij et al., 1997; Trainor et al., 2002), they also found increased activity in response to stimulus predictability in a number of frontal areas (medial-frontal gyrus, dorsal-prefrontal cortex, and superior-frontal gyrus). Many such studies have also found that musical training modulates activity patterns and connections between areas, illustrating the importance of previous experience, exposure and expertise in perception of rhythm and meter (Vuust et al., 2005, 2006; Chen et al., 2008a; Grahn and Rowe, 2009; Stupacher et al., 2013). These and other studies show a rhythm-related expertise-dependent action-perception reciprocity in the brain (Grahn and Brett, 2007; Chen et al., 2008b; Chapin et al., 2010; Kung et al., 2013), which may reflect the top-down/bottom-up mutuality and action-oriented perception posited by PC.

Neurophysiological research into rhythm and meter suggests similar mechanisms. Using EEG, Nozaradan et al. (2011) recorded neuronal entrainment during listening to a musical beat whose meter was imagined rather than manifested acoustically. Importantly, they found that properties of the beat which were only imagined (acoustically silent) elicited sustained oscillations tuned to the appropriate beat frequency, providing neural evidence of musical entrainment and induced meter. Fujioka et al. (2010) used MEG to show that listening to identical metronome clicks were spatiotemporally encoded in musicians’ brains in different ways, depending on the metric context according to which the ticks had been heard, either duple or triple. Specifically, the right hippocampus showed temporally differentiated peaks to both conditions, suggesting that this chiefly memory-related area may act as a predictor (or anticipator) during metric encoding of temporal structures. In the left basal ganglia, peaks corresponded to the duple condition only. As mentioned, duple meter is thought to be more salient than triple due to the inherent symmetry in human locomotion and, at least for certain populations, because of the bias toward duple meters in Western music. Therefore, the authors propose that the basal ganglia may be involved in the generation of metric hierarchies (duple is more salient than triple; Grahn and Brett, 2007; Grahn and Rowe, 2012). Finally, they speculate that the hippocampal memory system and striatal metric hierarchy system facilitate endogenous activation in auditory and auditory association areas through feedback loops. Studies such as these tap into the hierarchical yet dynamic nature of the brain’s functional organization at the millisecond level. Neurophysiological indications of entrainment, prediction, hierarchy and reciprocity in the brain are therefore highly compatible with the theory of PC.

Understanding the neural mechanisms underlying rhythm in a PC hierarchical framework has recently been suggested for the differential processing of timing at different time scales (Madison, in commentary to Clark, 2013). Whereas time representation at the level of milliseconds will typically be encoded close to the action output (e.g., cortical motor areas and the cerebellum), observations and actions that are more detached in time should involve more prefrontal processing. This is supported by studies showing processing distinctions between intervals above and below *circa* one second (Madison, 2001; Lewis and Miall, 2003; Gooch et al., 2011), as well as by indications that time representation for sub-second intervals are to some extent sensory specific (Nagarajan et al., 1998; Morrone et al., 2005) and under some conditions even limited to spatial locations (Johnston et al., 2006; Burr et al., 2007). For longer time periods, a larger part of the prefrontal cortex is activated (Lewis and Miall, 2006; Simons et al., 2006). This timing-related frontal lobe network overlaps with working memory and executive control networks (Jahanshahi et al., 2000; Owen et al., 2005), suggesting that timing constitutes a general cognitive control problem at longer time durations. As we shall see below, this division of labor persists in relation to the different time scales at which perceived rhythms can contradict the metrical framework. Whereas syncopations occurring at a single instance in drum rhythms with a clearly defined meter can be dealt with by the auditory cortices alone, polyrhythms that persist for several bars employ more frontally located (supposedly higher level) neuronal resources.

## NEURAL PROCESSING OF SYNCOPATION AND MUSICAL EXPERTISE

A key factor in our experience of rhythm is the extent to which a rhythmic pattern challenges our perception of meter. The most common example of such tension between rhythm and meter is *syncopation*. Syncopations are generally defined as rhythmic events which violate metric expectations (Longuet-Higgins and Lee, 1984; Fitch and Rosenfeld, 2007; Ladinig et al., 2009; Witek et al., in press). Generally, it is thought that listeners expect the majority of onsets in a rhythm to coincide with metrically salient positions, while rests or tied notes are expected to occur at metrically less salient positions (Longuet-Higgins and Lee, 1984; Temperley, 2010; Witek et al., 2014). A syncopation occurs when these expectations are violated, when onsets occur on metrically weak accents and rests or tied notes occur on metrically strong accents. Such expectations can be conceptualized in Bayesian terms (Temperley, 2007, 2010). The model assigns relative probabilities to all notes and rests of a pattern based on prior information about statistical frequencies and a hierarchical model of meter. A syncopation’s perceptual effect is thus a consequence of its predictability within the context of music as a whole. For a syncopation to obtain its characteristic effect, it must be experienced as contradicting the meter, but not so strongly that it undermines the meter. Syncopations can also be thought of as phase-shifts, where the rhythmic onset, rather than occurring in phase with its metric reference point, has a negative lag and occurs before it.

Auditory expectancy violations have been extensively studied via the “MMN” response in the brain (Sams et al., 1985; Näätänen et al., 1987, 2001; Paavilainen et al., 1989), a component of the auditory event-related potential (ERP), measurable with EEG and MEG. MMNs relate to change in different sound features, such as pitch, timbre, location of sound source, intensity, rhythm or other more abstract auditory changes, such as streams of ascending intervals (Näätänen et al., 1987, 2001; Näätänen, 1992; Friedman et al., 2001). The MMN is an effective way to measure pre-attentive prediction processes in the brain, and thus provides a particularly suitable tool to investigate PC. The MMN appears to have properties analogous to the error signal in a PC framework. It is dependent on the establishment of a pattern (or model) and responds only when the predictive pattern is broken. MMNs have been found in response to pattern deviations determined by physical parameters such as frequency (Sams et al., 1985), intensity (Näätänen et al., 1987), spatial localization, and duration (Paavilainen et al., 1989), but also to patterns with more abstract properties (Paavilainen et al., 2001; Van Zuijen et al., 2004). Importantly for our comparison with PC theories, the size of the MMN adjusts as the pattern adapts (Winkler et al., 1996), hence the size of the error message is dependent on the brain’s model of the incoming input as well as on the input itself.

The MMN is also strongly dependent on expertise. Musicians who adjust the tuning of their instruments during performance, such as violinists, display a greater sensitivity to small differences in pitch compared to non-musicians and other musicians playing other instruments (Koelsch et al., 1999); singers respond with a stronger MMN than instrumentalists to small pitch changes (Nikjeh et al., 2008); and conductors process spatial sound information more accurately than professional pianists and non-musicians (Münte et al., 2001). Recently, it was shown that performing musicians’ characteristics of style and genre influence their perceptual skills and their brains’ processing of sound features embedded in a musical context, as indexed by larger MMN (Vuust et al., 2012a,b). Such influences of training on low-level, pre-attentive neural processing exemplify the longer-term contextual, environmental and cultural aspects of PC.

To address the effects of expertise on metric perception, Vuust et al. (2009) investigated whether differential violations of the hierarchical prediction model provided by musical meter would produce error messages indexed as MMN. They compared rhythmically unskilled non-musicians with expert jazz musicians on two different types of metric violations: syncopations in the bass drum of a drum-kit (a musically common violation), and a more general (across all instruments of the drum-kit) disruption of meter (a musically less common violation). Jazz musicians frequently produce highly complex rhythmic music and are therefore ideal candidates for identifying putative competence-dependent differences in the processing of metric violations. MMNm (the magnetic equivalent to the MMN, measured with MEG) in response to metric disruption was found in both participant groups. All expert jazz musicians, and some of the unskilled non-musicians, also exhibited the P3am after the MMNm. The P3am is the magnetic equivalent of the P3a, an event-related response usually associated with the evaluation of salient change for subsequent behavioral action. The study also showed that responses to syncopation were found in most of the expert musicians. The MMNms were localized to the auditory cortices, whereas the P3am showed greater variance in localization between individual subjects. MMNms of expert musicians were stronger in the left hemisphere than in the right hemisphere, in contrast to P3ams showing a slight, non-significant right-lateralization.

The MMNm and P3am can be interpreted as reflecting an error term generated in the auditory cortex and its subsequent evaluation in a broader network of generators in the auditory cortex and higher-level neuronal sources. Consistent with this point of view is the fact that the MMN signal is mainly generated by pyramidal cells in the superficial layers of the cortex, as the canonical microcircuit implementation of PC suggests (Bastos et al., 2012). The study by Vuust et al. (2009) also showed indications of model adjustment in two of the jazz musicians, since their finger-tapping suggested a shift in metric framework (e.g., shifting of the position of the downbeat). These findings are thus in keeping with the PC theory and suggest that there is a congruous relationship between perceptual experience of rhythmic incongruities and the way that these are processed by the brain. However, PC is yet to determine the precise physiological localization and computations of the networks underlying such metric violations. Dynamic causal modeling (Stephan et al., 2007) is a relatively new neural network analysis tool that may help specify some of the unknowns in PC of rhythm and meter in music. Nonetheless, the study by Vuust et al. (2009) showed quantitative and qualitative differences in brain processing between two participant groups with different musical experience, indicating that prediction error generated by meter violation correlates positively with musical competence. A PC interpretation of these findings would posit that the metric models of musicians are stronger than those of non-musicians, leading to greater prediction error.

## PREDICTIVE CODING OF POLYRHYTHM

**FIGURE 3:**
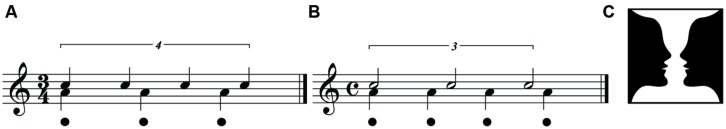
**Cross-rhythms. (A)** Three-beat triple meter with four-beat pattern as counter-rhythm. **(B)** Four-beat duple meter with three-beat counter-rhythm. Dots below the staves designate the tactus. **(C)** The bistable percept of Rubin’s vase.

In some styles of music, the meter may at times be only weakly (or not at all) acoustically actualized, a situation which creates extreme instances of perceptual rhythmic complexity. The pervasive use of *polyrhythm*, or even polymeter, throughout musical compositions is a radically complex rhythmic practice that occurs especially in (but is not restricted to) jazz music (Pressing, 2002). During polyrhythm the formal meter may be completely absent in the actual acoustic signal, and musicians must rely on listeners’ ability to predict the formal metric framework. One example of polyrhythm is “cross-rhythm,” in which different overlaid rhythmic patterns can be perceived as suggesting different meters (Danielsen, 2006). A typical example is the so-called “three-against-four” pattern, which may be illustrated by tapping three equally spaced beats in one hand and four equally spaced beats in the other at the same time, so that the periods of both patterns are synchronized. It is possible to perceive the meter of such a pattern in two ways, either as triple or duple. In triple meter, the formal time signature is 3/4 and the four-beat pattern acts as a counter-metric pattern (**Figure 3A**). In duple meter, the time signature is 4/4 and the three-beat pattern is the counter-metric pattern (**Figure 3B**). The rhythmic organization of the two interpretations in **Figure 3** is exactly the same; that is, in each pattern the cross-rhythmic relationship between the two streams is identical. The pattern notated in the lower part of the staves expresses the meter while the pattern in the higher part is the counter-rhythm. The phenomenological experience of this polyrhythm therefore depends on which of the patterns in the cross-rhythm is defined as the meter. The three-against-four polyrhythm is thus analogous to ambiguous images such as Rubin’s vase, which can be seen either as a vase on black background, or faces on white background (**Figure 3C**). In the case of the cross-rhythms, the meter is the background and the counter-metric rhythm is the foreground. As with Rubin’s (1918) vase, cross-rhythm in music can sometimes cause perceptual shifts in which the metric model can be reinterpreted in a different way. In music, such metric shifts can be supported by sensorimotor synchronization, e.g., foot-tapping emphasizing the tactus of the meter. Phillips-Silver and Trainor (2005) found that after an initial period of listening to metrically ambiguous rhythms while being bounced according to either a duple or triple meter, 7-month old babies preferred (i.e., listened longer to) rhythms with accent patterns (i.e., meter) to which they had previously been bounced. Similar patterns were found in adults, suggesting that auditory and vestibular information affects rhythm and meter perception (Phillips-Silver and Trainor, 2007, 2008). Viewed as PC, their findings indicate that body-movement shapes perception, suggesting action-oriented perception (Clark, 2013). Polyrhythms and otherwise ambiguous rhythms can thus be seen as presenting to the listener a bistable percept (Pressing, 2002) that affords rhythmic tension and embodied engagement.

Bistable percepts and other types of perceptual illusions have been suggested to provide particularly revealing illustrations of PC (Hohwy et al., 2008; Clark, 2013). A common example is binocular rivalry, a perceptual scenario in which, using a special experimental setup, each eye is shown a different image simultaneously – for example, a house and a face (Leopold and Logothetis, 1999; Hohwy et al., 2008). In such experiments, the experienced image is not a combination of the two images – some morphed structure with both house- and face-features – but rather a bistable percept in which the image shifts from one to the other, but never the two at the same time. According to PC, such artificially induced experiences illustrate how our perceptual system deals with situations in which there are more than one predictive model. The bottom-up input presents two equally plausible models – it is just as common to see a house as it is too see a face – but they are temporally and spatially incompatible, i.e., the hyper-prior is that we never see a face and a house as coming from the same source at the same time. However, no one stable model can be said to be more likely or more expected than the other. In choosing one hypothesis over the other, the top-down signals will “explain away” only those elements of the driving signal that conform to this hypothesis, causing the prediction error of the alternative hypothesis to be forwarded upward in the system. Therefore, no single prediction can account for all the incoming information or reduce all prediction error, and the brain alternates between the two semi-stable percepts. While non-Bayesian feed-forward accounts of such scenarios posit that switching is caused by attention alone (e.g., Lee et al., 2005), PC posits a “top-down” competition between linked sets of hypotheses.

In a similar way, we can perceive two alternative rhythms in cross-rhythmic patterns of the kind depicted in **Figure 3**, but never both at the same time. In such complex cases, perceptually alternating and prediction-switching processes are the best way for the brain to minimize prediction error and maintain a statistically viable representation of its environment. However, cross-rhythmic patterns differ from binocular rivalry in one important way: in cross-rhythms such as the three-against-four pattern, it is possible for musically trained individuals to consciously “hear” one interpretation of the pattern, despite the perceptual input advocating for the other. In such cases, the perceiver must devote considerable effort to sustain his or her internal metric model while the rhythmic input deviates from it.

**FIGURE 4:**
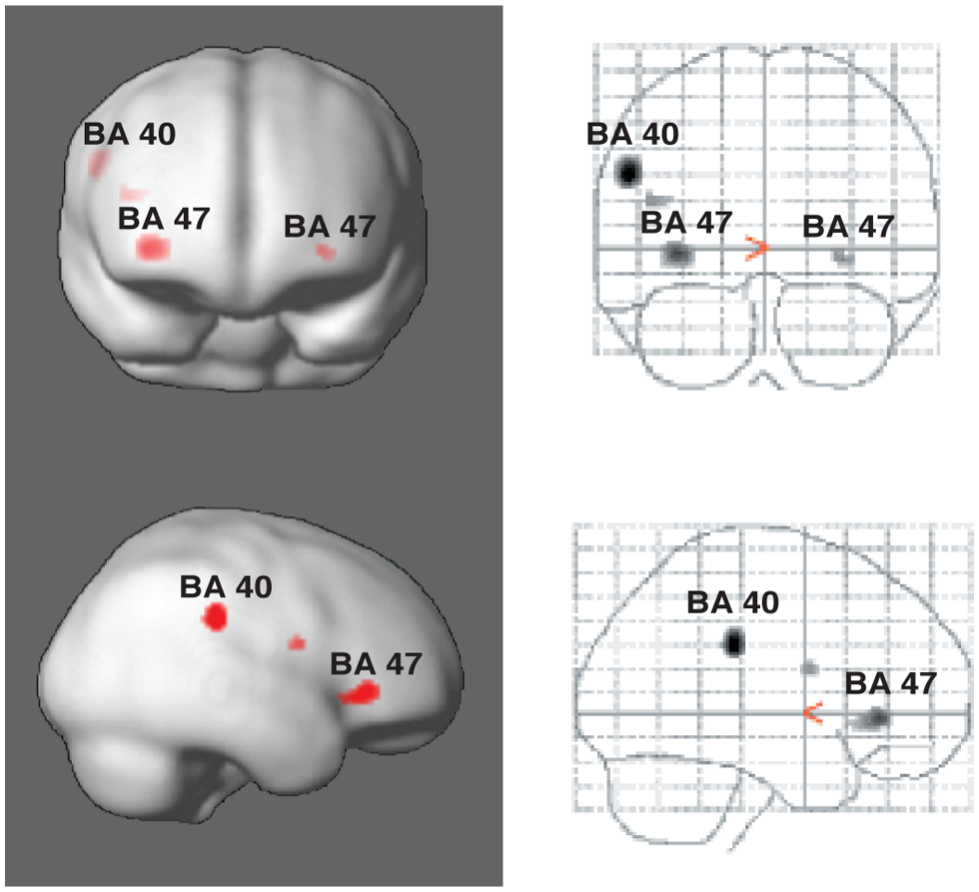
**Areas of activity in the brain during tapping to polyrhythm.** Activations of Brodman’s areas (BA) 40 and 47 in the parietal and prefrontal cortices, respectively, as associated with tapping to polyrhythms. See Vuust et al. (2006) for more detail.

Vuust et al. (2006, 2011) have taken advantage of these alternative perceptual consequences of polyrhythm in two studies, using fMRI to measure blood-oxygenated-level-dependent (BOLD) responses in “rhythm section” musicians (drummers, bassists, pianists, and guitarists). The musical example used was the soprano saxophone solo in Sting’s “Lazarus Heart,” in which the rhythm suddenly changes to a different meter for six measures, leaving no acoustic trace of the original meter. However, despite the shift in the musical surface, it is still possible to infer the original meter since the subdivisions and metric frameworks of the two eventually align at the end of the six measures. In other words, a listener could, depending on his or her musical-temporal abilities, consciously maintain the counter-meter. During the first experiment, participants were asked to tap along to the main meter of the music while mentally focusing first on the main meter and then on the counter-meter (Vuust et al., 2006, 2011). In the second experiment, they listened to the main meter throughout the study and were asked to tap both the main meter and the counter-meter. In the second experiment, it was found that Brodman’s area (BA) 40 showed increased activity during tapping to the counter-meter compared to the original meter (**Figure 4**). This brain area has been associated with language prosody, and with particular relevance for our discussion, with bistable percepts (Kleinschmidt et al., 1998; Lumer et al., 1998; Sterzer et al., 2002). Furthermore, in both experiments, the counter-metric tasks showed increased activity in a part of the inferior frontal gyrus corresponding to BA 47, most strongly in the right hemisphere (**Figure 4**). This area is typically associated with language, particularly semantic processing (for reviews, see Fiez, 1997; Cabeza and Nyberg, 2000). Vuust et al.’s (2006, 2011) studies thus suggest that these areas may serve more general purposes than formerly believed, such as sequencing or hierarchical ordering of perceptual information (BA 47) (Fiebach and Schubotz, 2006), and predictive model comparisons (BA 40). Interestingly, BA 47 was found to be active both in relation the experience (experiment 1) and production (experiment 2) of polyrhythmic tension. Therefore, it is possible that this area, bilaterally, is involved in the processing of prediction error in polyrhythm *per se*. The findings may thus provide evidence of action-oriented predictive processing and the close relationship posited between action and perception in PC (Clark, 2013). Furthermore, activity in BA 47 was inversely related to rhythmic expertise as measured by standard deviation of finger-tapping accuracy. In other words, the effort to maintain a counter-metric model during polyrhythm requires less brain activity for musicians than for non-musicians. This finding supports the PC hypothesis that the more accurate the prediction, the less processing is needed by the perceptual system. According to PC, the continuous effort needed to sustain a counter-metric model should lead to sustained activity in the relevant brain areas (e.g., BA 47) and networks, including areas at higher levels than those primarily generating the prediction errors. At these higher levels, the experts’ models should be more successful at predicting the incoming rhythmic information since they require less “processing power” to maintain a competing metric model. In this way, the decreased neural activity in response to increased musical ability in expert musicians is an expression of the hierarchical, bidirectional, and context-sensitive mechanisms posited by PC.

## PREDICTIVE CODING IN GROOVE

In certain styles of music, such as funk (Danielsen, 2006), hip-hop (Greenwald, 2002) and electronic dance music (Butler, 2006), continuous rhythmic complexity is the basis for structural development. Such music is often referred to as “groove-based” (Danielsen, 2010). Groove is primarily defined as a psychological construct, characterized by a pleasurable drive toward body-movement in response to rhythmically entraining music (Madison, 2006; Madison et al., 2011; Janata et al., 2012; Stupacher et al., 2013; Witek et al., 2014). Such behavioral effects require that the rhythmically complex musical structures, such as syncopation and cross-rhythm, are continuously repeated. Other examples of repeated rhythmic complexity in groove are metric displacement (Butler, 2006; Danielsen, 2006) and microtiming (Waadeland, 2001; Iyer, 2002; Danielsen, 2006).

In recent experiments, Witek et al. (2014) investigated the relationship between syncopation in groove, the desire to move, and feelings of pleasure. Their stimuli consisted of 50 groove-based (funk) drum-breaks, in which two-bar rhythmic phrases featuring varying degrees of syncopation were repeated four times, continuously. Using a web-based survey, participants were asked to listen to the drum-beaks and rate the extent to which they felt like moving and experienced pleasure. The results showed an inverted U-shaped relationship between degree of syncopation and ratings, indicating that intermediate degrees of rhythmic complexity afford optimal pleasure and desire for body-movement. The inverted U is a familiar function (Wundt, 1874) in music psychology (North and Hargreaves, 1995; Orr and Ohlsson, 2005) and has been suggested to describe the relationship between perceptual complexity and arousal in art more broadly (Berlyne, 1971). Interestingly, rather than being affected by participants’ formal musical training, Witek et al. (2014) found that those who enjoyed dancing and often danced to music rated the drum-breaks as eliciting more pleasure and more desire to move, overall. Thus, it seems that not only institutionalized formal musical training, but also more informal embodied experience with music may affect subjective experiences of rhythmic complexity such as groove.

The inverted U-shape found between degree of syncopation in groove, wanting to move, and feelings of pleasure can be seen as complying with a hierarchical perceptual system at its higher and more subjectively manifested levels. At this higher level, prediction in perception and action facilitates affective and embodied experiences. At low degrees of syncopation, there is little incongruence between the rhythm of the groove (the input) and the meter (the predicted metrical model). Thus, little prediction error is being fed forward from the lower to the higher levels, and the experiential effect is weak – there is little pleasure, and little desire to move. At high degrees of syncopation, the degree of complexity is so high, and the rhythmic input deviates from the metric framework to such an extent, that the predicted model breaks down. Affective and embodied responses are decreased since the system is in the process of “learning” and adjusting its internal models. Also here there is little prediction error since the brain is unable to provide an appropriate prediction model to compare the incoming input with. This uncertainty of the system in the initial phase of perception is widely reported in the literature (Pack and Born, 2001; Born et al., 2010) and is what one would expect if perception involved recruiting top-level models to explain away sensory data. However, at intermediate degrees of syncopation in groove, the balance between the rhythm and the meter is such that the tension is sufficient to produce prediction error, and for the perceptual system to come up with a prediction model, but not so complex as to cause the metric model to break down. The input and the model are incongruent, but not incompatible, and the prediction error affords a string of hierarchical encoding and evaluation from lower to higher levels in the brain, ultimately facilitating feelings of pleasure and desire to move. In fact, synchronized body-movement in groove-directed dance is a good example of action-oriented perception, since the body essentially emphasizes the predictive model by moving to the beat and hence actively tries to minimize prediction error.

These nested levels of input-model comparisons can also explain why it is that, despite persistent repetition, the rhythmic complexity in groove does not lose its characteristic perceptual effect. That is, higher levels in the groove processing hierarchy do not only provide basic perceptual metric models (i.e., rhythmic onsets should occur on strong and not weak accents), they also model expected deviations from the meter (i.e., in groove, rhythmic onsets often occur on metrically weak accents). In this way, groove remains complex, and there is constant tension between rhythm and meter, despite the same rhythmically complex patterns being repeated time and time again. The relationship between lower and higher models can thus be one of tension itself.

Prediction and expectation have been proposed as the primary mechanisms for emotion and pleasure in music (Meyer, 1956; Huron, 2006). The general idea in Huron’s theory is that the brain rewards behavior that stimulates prediction, since prediction is an evolutionarily adaptive cognitive ability. However, it should be noted that although PC has been claimed to provide a “grand unifying theory” of cognition and brain processing, able to provide explanations from low-level firing in individual neurons to high-level conscious experience, perceptual inputs are of course not necessarily evaluated and consciously perceived in terms of prediction (Clark, 2013). That is, when we listen to groove-based music, we may not be consciously perceiving violations of expectation and prediction errors. Of course, in experience, affective and embodied responses are more readily available to evaluation. Rather, PC should be seen as the system “working in the background” to facilitate the characteristic affective and embodied experiences with groove.

This discussion highlights how music and the relationship between rhythm and meter can illustrate the PC theory. The apparent paradox of the pleasure felt in relation to moderate amounts of syncopation is an example of the so-called “dark room problem,” which was recently highlighted in Schaefer et al.’s comment to Clark’s paper, and his subsequent response (Schaefer et al., in commentary to Clark, 2013). What is clearly consistent with PC is that the prediction error between the meter representation and the syncopated rhythm depends on the brain’s ability to infer a meter. Rhythm with low syncopation should only entail small or no prediction errors related to the meter. Rhythm with medium syncopation, i.e., still possible for the brain to reconcile with a certain metric interpretation, will lead to larger prediction errors. Rhythm with too much syncopation, however, could lead to less prediction error if the brain cannot find the meter, even though the complexity in the stimulus is objectively greater. In other words, there cannot be an increase in prediction error if there is no model to compare the input with. What is not evident is why prediction error could lead to higher experience of pleasure. The “dark room problem” in this situation is how to bridge the gap between neuronal activity and organization, and conscious and subjective experience.

Clark addresses this problem by stating that the brain’s end goal is to maximize prediction, rather than minimize prediction error. Thus, the brain may be rewarding prediction error since it leads to learning (i.e., maximizing future prediction). A likely candidate for mediating this effect is the neurotransmitter dopamine in the mesolimbic pathway, as suggested by Gebauer et al. (2012). Research in rodents (Schultz, 2007; Schultz et al., 2008) has shown dopamine release to both expected and unexpected stimuli, suggesting that the complex interaction between dopamine release and predictions ensures a balance between “explaining away” prediction error in the short term, and maintaining an incentive to engage in novel activities (of potential high risk) leading to adaptive learning in the long term. A next step would be to empirically validate whether the relationship between syncopation in groove and pleasure is modulated by the dopamine system, and to what extent prediction describes the underlying system at both behavioral and neural levels.

## CONCLUSION

The hierarchical nature of meter and the relationship between rhythm and meter in rhythmic complexity provide particularly suitable examples of predictive coding in music. Predictive coding posits that perception and action are mechanisms relying on hierarchical processing of information in Bayesian terms, by which perceptual input, modulated by motor action, is compared with predictive models in the brain. In music, rhythm (the input) is heard in relation to meter (the model). When these are at odds, the difference between them (the prediction error) is fed forward into the system and is subjected to a string of computational evaluations at each level of the perceptual hierarchy, from low-level neuronal firing to high-level perception and cognition. The predictive models are inferred from previous experience, and thus the system is always in a relationship between bottom-up and top-down processes. We suggest that during syncopation – a rhythmic structure that violates metric expectations – the listener’s previous musical training determines the strength of the metric model, and thus the size of the prediction error. Polyrhythm is a type of bistable percept in the auditory domain, which relies on competition between different predictive models to achieve its perceptually characteristic effect. In groove, medium degrees of syncopation provide the optimal balance between prediction and complexity, allowing for just enough prediction error to stimulate the cascade of model comparisons at the nested levels of the perceptual hierarchy and elicit the characteristic pleasurable desire to dance. Further, the constantly repeated rhythmic complexity in groove resists permanent model shifts of low-level metric frameworks, because higher-level models predict that groove should be complex. These instances of rhythmic complexity in music thus provide unique examples of several different properties of predictive coding, and present us with ecologically valid stimuli to use in studying human perception, action, prediction, and the brain.

## Conflict of Interest Statement

The authors declare that the research was conducted in the absence of any commercial or financial relationships that could be construed as a potential conflict of interest.

## Footnotes

1 More levels can be defined above the bar-level (e.g., the hyper-bar level) and below the 16th level (e.g., the 32nd and 64th levels). Theoretically, metric levels are relative and can be subdivided indefinitely. In practice, however, the metric levels we perceive are limited by our perceptual system: Time-spans too long or too short are not detectable to the human ear (Fraisse, 1984).
